# Dental-derived stem cells in tissue engineering: the role of biomaterials and host response

**DOI:** 10.1093/rb/rbad100

**Published:** 2023-11-10

**Authors:** Weihao Yuan, Luiza de Almeida Queiroz Ferreira, Bo Yu, Sahar Ansari, Alireza Moshaverinia

**Affiliations:** Weintraub Center for Reconstructive Biotechnology, Section of Prosthodontics, School of Dentistry, University of California, Los Angeles, Los Angeles, CA 90095, USA; Weintraub Center for Reconstructive Biotechnology, Section of Prosthodontics, School of Dentistry, University of California, Los Angeles, Los Angeles, CA 90095, USA; Department of Restorative Dentistry, School of Dentistry, Universidade Federal de Minas Gerais, Belo Horizonte, Minas Gerais, Brazil; Section of Restorative Dentistry, School of Dentistry, University of California, Los Angeles, Los Angeles, CA 90095, USA; Weintraub Center for Reconstructive Biotechnology, Section of Prosthodontics, School of Dentistry, University of California, Los Angeles, Los Angeles, CA 90095, USA; Weintraub Center for Reconstructive Biotechnology, Section of Prosthodontics, School of Dentistry, University of California, Los Angeles, Los Angeles, CA 90095, USA; Department of Bioengineering, Henry Samueli School of Engineering and Applied Sciences, University of California, Los Angeles, Los Angeles, CA 90095, USA

**Keywords:** dental-derived stem cells, biomaterials, host response, tissue engineering

## Abstract

Dental-derived stem cells (DSCs) are attractive cell sources due to their easy access, superior growth capacity and low immunogenicity. They can respond to multiple extracellular matrix signals, which provide biophysical and biochemical cues to regulate the fate of residing cells. However, the direct transplantation of DSCs suffers from poor proliferation and differentiation toward functional cells and low survival rates due to local inflammation. Recently, elegant advances in the design of novel biomaterials have been made to give promise to the use of biomimetic biomaterials to regulate various cell behaviors, including proliferation, differentiation and migration. Biomaterials could be tailored with multiple functionalities, e.g., stimuli-responsiveness. There is an emerging need to summarize recent advances in engineered biomaterials-mediated delivery and therapy of DSCs and their potential applications. Herein, we outlined the design of biomaterials for supporting DSCs and the host response to the transplantation.

## Graphical Abstract

**Figure:**
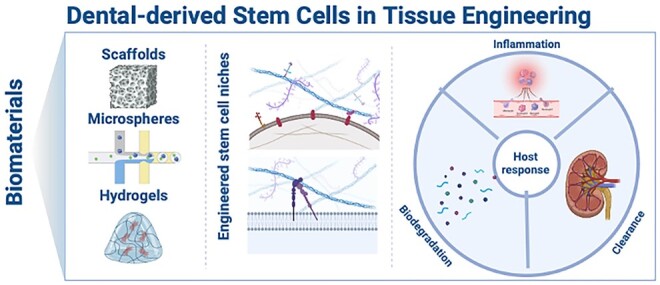

## Introduction

Dental-derived cells, especially those from dental pulp and periodontal ligament, can differentiate into various functional cells. These stem cells have significant potential for regenerative medicine and tissue engineering [1–3]. Dental stem cells (DSCs) are a subset of adult stem cells capable of transforming into various cell types, such as bone, cartilage, muscle and fat cells [4–6]. Owing to their regenerative capabilities, DSCs hold substantial potential for repairing or regrowing tissues afflicted by disease or damage, making them a subject of significant interest within the domain of tissue engineering [7]. A pivotal rationale for applying DSCs in tissue engineering is their capacity to transform into numerous cell types. This versatility enables their utilization in various tissue engineering implementations [8, 9]. DSCs have an easily accessible source, compared with bone marrow/adipose-derived MSCs, which are most extensively studied in current research, facing a severe shortage of donors because the harvest of bone marrow/adipose-derived MSCs requires invasive surgical procedures [10]. Other limitations include the complicated and long-term separation and culture of bone marrow/adipose-derived MSCs due to the deficient population of MSCs in bone marrow and adipose tissue and loss of proliferation and multilineage differentiation capacity [11]. DSCs, which can be extracted from exfoliated deciduous teeth or discarded dental tissues, hold great promise to serve as an alternative cell source for tissue engineering [12–14].

DSCs can generate and secret various bioactive factors that can stimulate the growth and regeneration of multiple tissues [15, 16]. In addition, DSCs have a solid ability to migrate to areas of tissue damage and inflammation, which makes them useful for tissue regeneration. The immunomodulatory properties of DSCs, including MSCs, have been identified to play a crucial role in tissue regeneration. Multiple DSCs can secret substantial immunoinhibitory cytokines, including TGF-β and IL-10 [17, 18]. Moreover, this process can be enhanced by other immunomodulatory cytokines, especially the cytokines secreted by macrophages and T cells [19, 20]. Compelling evidence supports the idea that successful tissue regeneration via stem cells hinges on the critical interaction between DSCs and immune cells. Additionally, DSCs have been proven to possess many immunomodulatory effects, notably the capacity to inhibit the activation of immune cells [21].

The ECM is a sophisticated lattice comprising proteins, glycoproteins, glycosaminoglycans and various other molecules, collectively providing a supportive milieu for the cells in tissues [22]. It acts as a cellular scaffold and facilitates cell communication, tissue structure and repair. Upon encountering the ECM, DSCs can detect and react to its myriad physical and biochemical cues [23, 24]. This can influence the behavior of DSCs, including their differentiation, migration and survival. For example, DSCs may differentiate into different cell types based on the specific ECM proteins they encounter, and they may migrate toward areas of tissue damage or inflammation in response to ECM signals. In brief, the intricate relationships between DSCs and the ECM are essential for the regeneration of tissues. By adhering to the ECM and secreting its proteins, DSCs can shape tissue structure and function and, thus, actively participate in the healing and regeneration of injured tissues [25–27].

Direct MSC transplantation to damaged tissues may face problems in pilot trials, such as low retention and survival rate in target sites, poor differentiation into specific mature tissues, and severe local inflammation [28, 29]. Biomaterials are materials designed to interact with living tissues and can be used to support or replace damaged or diseased tissues. Biomaterials can be natural or synthetic and can be used in various forms, including scaffolds, hydrogels and microspheres. Combining biomaterials with DSCs in tissue engineering can protect the DSCs and keep them in place until they can integrate into the surrounding tissue, providing a supportive environment for DSCs [30, 31]. The combined effects of DSCs and biomaterials have led to substantial advancements in tissue engineering, showcasing the potential to treat diverse conditions such as inflammatory diseases, tissue injuries and degenerative disorders. Hence, it is increasingly essential to consolidate the current progress in applying dental-derived materials for tissue engineering, mainly focusing on the roles of biomaterials and the host response.

This review summarizes the revolution of biomaterials and the interactions between DSCs and biomaterials. We then classify the host response to the transplantation of biomaterials. We hope this review can illuminate the design of novel biomaterials in tissue engineering.

## Design principles of biomaterials and interactions between DSCs and biomaterials

### Classification of biomaterials in tissue engineering

Biomaterials used in tissue engineering can be classified in a variety of ways, including by their origin (natural or synthetic), their physical properties (such as their mechanical strength or biodegradability) and their intended use (such as scaffolds, hydrogels or microspheres) [32, 33].

One common way to classify biomaterials is by their origin. Natural biomaterials are derived from living organisms or their components, while synthetic biomaterials are made entirely from nonbiological materials. Natural biomaterials include collagen, chitosan and silk, while synthetic biomaterials include polyethylene, polystyrene and polyethylene terephthalate [34–36]. Another way to classify biomaterials is by their physical properties. Biomaterials can be classified based on their mechanical strength, biodegradability and physical characteristics. For example, some biomaterials are solid and durable, while others are designed to be biodegradable and able to be absorbed by the body [37–39].

**Figure 1.**
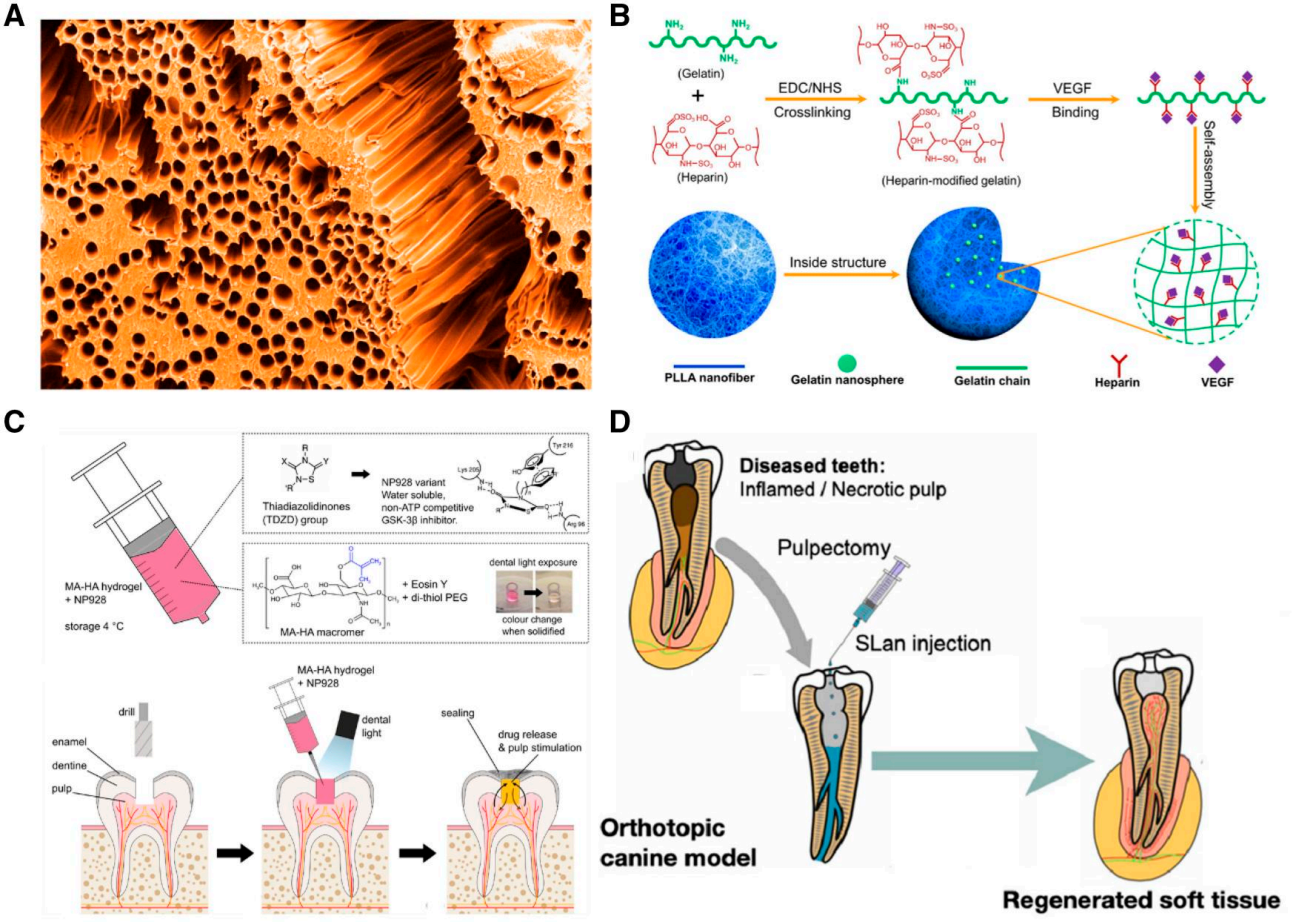
Design principles of biomaterials and interactions between DSCs and biomaterials. (**A**) The cross-sectioning SEM image of the microstructures of tubular scaffolds. Reproduced with permission from Ref. [43], Copyright 2014, Elsevier. (**B**) Schematic illustration of the synthesis and fabrication of microsphere. Reproduced with permission from Ref. [47], Copyright 2016, Elsevier. (**C**) The design of methacrylate hyaluronate-based hydrogel combined with NP928 drug. Reproduced with permission from Ref. [51], Copyright 2022, International & American Associations for Dental Research. (**D**) The fabrication of peptide-based self-assemble hydrogel for dental tissue engineering. Reproduced with permission from Ref. [52], Copyright 2021, Elsevier.

Significantly, biomaterials can also be categorized according to their specified application in tissue engineering. Scaffolds, for instance, are three-dimensional constructs providing a framework for cellular growth, which can be employed in the repair or regeneration of tissues [40–42]. Haeri and Goldberg [43] developed an acrylate-based microtubular scaffold to mimic the structure of natural dentin. The scaffold was made by the templating method, in which sacrificial polyvinyl alcohol fiber was placed inside the acrylate monomer, followed by the polymerization and leaching process. The obtained scaffold can effectively support the differentiation of DSCs and the formation of mineralized dentin tissue (Figure 1A). Microspheres are small, spherical particles that can deliver drugs or cells to specific locations in the body [44–46]. Liu *et al.* reported a vascular endothelial growth factor (VEGF) encapsulating poly (l-lactic acid) microsphere (Figure 1B). The discussed microsphere has been said to continuously release VEGF, promoting the growth and differentiation of dental cells to facilitate the regeneration of dental pulp [47]. Hydrogels are highly hydrated polymer networks that can deliver cells or drugs to specific locations in the body [48–50]. Sharpe *et al.* [51] reported a methacrylated hyaluronate hydrogel loaded with GSK3 inhibitor NP928 to regenerate dentin. The loaded NP928 drug can effectively promote the activity of the Wnt/β-catenin signaling pathway, thereby promoting the differentiation of DSCs into mineralized dentin tissue (Figure 1C). Yang *et al.* [52] developed a peptide-based self-assemble hydrogel for dental pulp regeneration. The top-left panel of Figure 1D showed the diseased teeth with inflamed/necrotic pulp, in which the blood vessels were severely damaged. After the injection of self-assembled hydrogels, the bottom-right panel of Figure 1D showed the significant neovascular formation in the regenerated pulp (Figure 1D). Overall, the classification of biomaterials in tissue engineering can vary depending on the specific characteristics and intended use of the materials. Understanding different biomaterials’ properties and potential benefits can help researchers design effective therapies for various conditions.

### Engineered biomaterials mimicking the interactions between DSCs and natural ECM

In tissue engineering, designed biomaterials that replicate the interactions between DSCs and the ECM can be utilized to aid the repair and regeneration of damaged tissue. The ECM is a sophisticated web of proteins and other molecules that encapsulates and supports cells within tissues, playing an integral part in upholding tissue structure and functionality [53–55]. DSCs engage with the ECM via integrin receptors located on their surface. These integrins are transmembrane proteins that bind to specific ECM proteins, like collagen and fibronectin. Such binding enables DSCs to anchor themselves and receive signals that regulate their behaviors, including their proliferation, differentiation and migration [56–58].

Engineered biomaterials that mimic the ECM can be designed to provide a similar environment for DSCs to interact with to support their differentiation, migration and survival. The emulation of engineered biomaterials on natural ECM offers a supportive microenvironment for DSCs. Biomaterials can be leveraged to fabricate scaffolds or similar structures that emulate the physical and biochemical characteristics of the extracellular matrix (ECM). This facilitates the differentiation of DSCs into functional cells, vital for tissue engineering and regenerative medicine, while shielding them from local inflammation [59–61]. Modifying the natural ECM’s stiffness necessitates adjusting matrix protein concentration, leading to increased ligand density and decreased mesh size [62]. In contrast, the rigidity of synthetic biomaterials can be changed without altering ligand density, significantly aiding mechanobiology studies on cells encapsulated in a 3D matrix [63–65]. Therefore, material and biological scientists have gained increasing attention from engineered biomaterials, specifically supramolecular interaction-crosslinked hydrogels.

**Figure 2.**
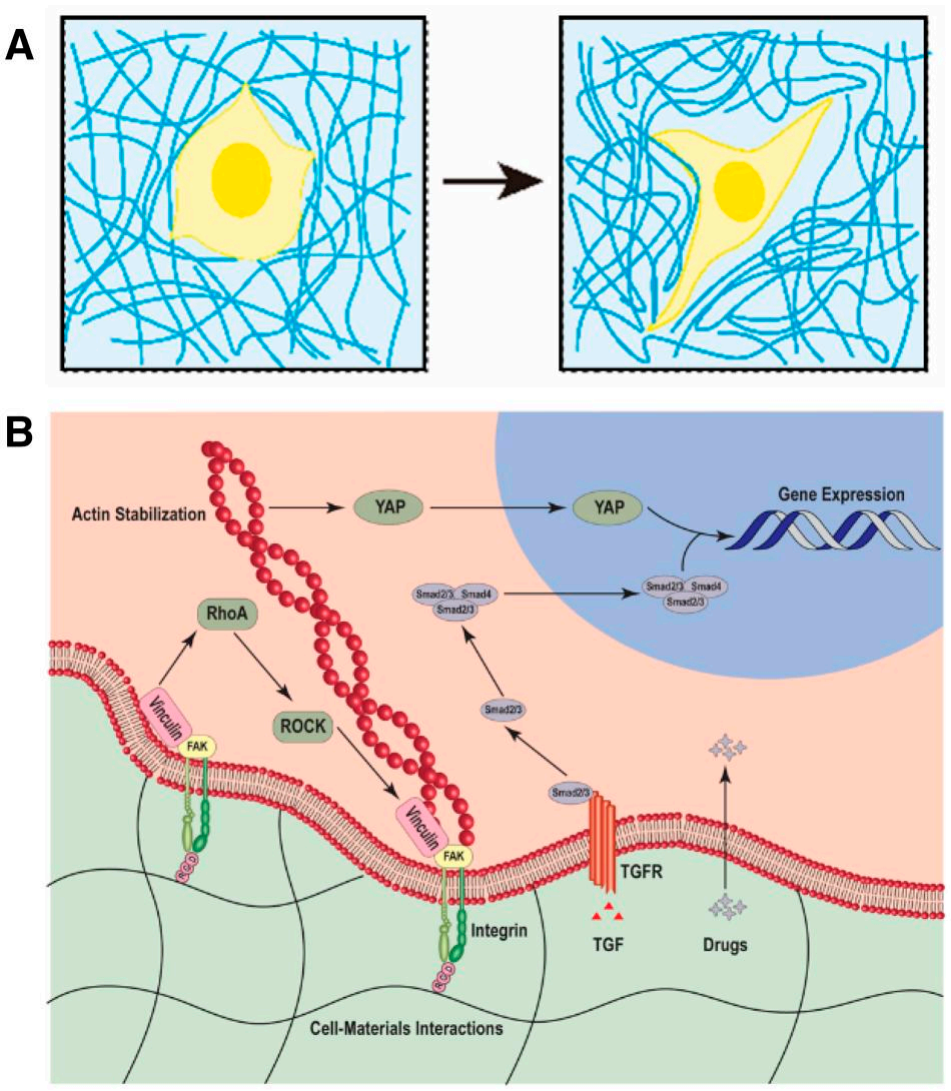
Schematic illustration of the interactions between DSCs and biomaterials. (**A**) The matrix remodeling via the cell traction force-induced deformation. (**B**) The mechanism of biomaterials-mediated determination of DSC fate: the activation of mechanoresponsive signaling proteins via transduced mechanics of the ECM, the interactions between cytokines and their receptors (e.g., TGF family) and loaded drugs.

Supramolecular hydrogels, with their unique viscoelastic properties, have been found to regulate various cell behaviors, including proliferation, differentiation, migration and invasion, and they can also sustain multiple stem cells for long-term tissue regeneration [66, 67]. A broad assortment of integrins act as the central receptors associated with mechanosensing. They can relay the pressures the ECM imposes from cell membranes to numerous intracellular components [68–70]. Considering the distinct characteristics of supramolecular hydrogels and the mechanosensing-related signaling pathways in stem cells, these hydrogels can serve as 3D synthetic stem cell niches. These niches can modulate cellular behavior and steer cell destiny [71–73]. The dynamics of the hydrogel assist in reshaping the matrix through deformation induced by cell traction forces, thus enabling the cells to proliferate, spread out and transform following the mechanics of the engineered matrix (Figure 2A). When subjected to specific biochemical or mechanical stimuli from carefully constructed stem cell niches, stem cells can be programmed to differentiate into cell types or to organize into distinct tissue forms autonomously [69, 74, 75]. The relayed mechanical aspects of the ECM can trigger the activation of mechanoresponsive signaling proteins such as focal adhesion kinase, microtubule-associated protein kinase, Ras homolog gene family member A and YAP/TAZ signaling. Alternatively, they can establish a direct connection with the nucleus to oversee the epigenetic transcription of chromatin [63, 76, 77] (Figure 2B). Furthermore, the determination of DSC fate can be achieved through interactions between cytokines and their receptors (e.g., TGF family) and incorporated drugs.

The potential of engineered biomaterials that simulate interactions between DSCs and the ECM for tissue engineering is substantial, specifically in repairing and regenerating damaged tissues. However, more research is warranted to fully comprehend these biomaterials’ most effective properties and designs to harness their capabilities for tissue repair and regeneration.

### Stimuli-responsive properties of engineered biomaterials

Stimuli-responsive biomaterials are specifically crafted materials capable of altering their physical or chemical characteristics in reaction to specific triggers like temperature, pH, or light. The potential applications of these materials span a wide range, including tissue engineering, drug delivery and biosensing.

In tissue engineering, biomaterials that react to stimuli can furnish a more dynamic and adaptable environment for cellular growth and differentiation. For instance, a temperature-responsive biomaterial could offer varied cell backgrounds based on temperature, subsequently impacting cell behavior. Biomaterials responding to pH or light can deliver therapeutic cells and drugs. He *et al.* [78] employed a nanocomposite hydrogel for tissue regeneration. By integrating the antibiotic moxifloxacin hydrochloride and stem cells, the nanocomposite hydrogel became pH-responsive and could precisely deliver and release the drug and stem cells in an acidic microenvironment. Han *et al.* [79] crafted a light-responsive nanocomposite hydrogel composed of PNIPAM and PDA NPs, which underwent phase transitions and volume changes under near-infrared (NIR) light. This enabled NIR light to induce drug and stem cell release and facilitate healing, thereby serving the diverse needs of biomedical applications.

### Translational potential of biomaterials

Biomaterials possess an extensive array of potential medical uses, encompassing tissue engineering, drug delivery and medical implants. Nevertheless, the transition of biomaterials from research labs to clinical settings necessitates rigorous testing to validate their safety and effectiveness [80]. A critical aspect of the translational potential of biomaterials is their biocompatibility, which refers to their ability to interact with biological systems without causing an adverse response. For medical applications, biomaterials must be biocompatible, nontoxic, noninflammatory and nonimmunogenic. Another critical factor in the translational potential of biomaterials is their ability to be manufactured at scale and at a reasonable cost. Biomaterials that are difficult or expensive to manufacture may not be practical for clinical applications [81]. Some biomaterials may be classified as medical devices, with a shorter approval process than drugs or biologics. However, even medical devices may require significant testing and documentation to demonstrate safety and efficacy.

## Host response to the transplantation of biomaterials

### Effects of cell-laden engineered biomaterials on local inflammation

Local defects or damaged tissues can initiate the release of a series of damage-associated molecular patterns, which will recruit various immune cells and activate the release of inflammatory cytokines, thereby causing severe local inflammation [82–86]. The ability of DSCs to modulate the immune response positions them as a promising therapeutic choice for an array of conditions, such as inflammatory diseases, tissue damage and degenerative disorders. Commonly utilized in tissue engineering and regenerative medicine for repairing and regrowing damaged tissue, DSCs are also under investigation for their potential application in treating autoimmune diseases and combating transplant rejection.

The immunomodulatory properties of DSCs can be mediated by various signaling, including cytokines, chemokines and growth factors [87–89]. These molecules can regulate the immune response and mitigate inflammation by restraining immune cells’ activation and growth, including T and B cells [90, 91]. Nishimura *et al.* [92] demonstrated that DSCs could promote the polarization of macrophages to M2 phenotype and prevent periodontal bone loss through paracrine. More precisely, they can discharge extracellular vesicles (EVs) that house immunoinhibitory miRNAs and proteins, subsequently restraining the release of inflammatory cytokines from immune cells and promoting polarization towards prohealing subtypes. Additionally, they can spur the formation of regulatory T cells, which are beneficial in dampening immune responses and lessening inflammation. Zheng *et al.* found that the miRNAs in the exosomes secreted by DSCs can change the balance of Th17 and Treg cells and reverse the immune imbalance in periodontitis. The delivery of DSCs and their EVs via engineered biomaterials holds great therapeutic potential in tissue engineering and regenerative medicine. It identified a series of candidate biomolecules in EVs, which can significantly promote tissue regeneration or inhibit local inflammation. Although it is hard to get FDA approval for the combinational delivery of DSCs and EVs, the identified candidate biomolecules in EVs, including miRNAs, cytokines and enzymes, can be synthesized *in vitro* and used for synergistic therapy with DSCs.

Engineered biomaterials can be deliberately designed to work with DSCs to further control local inflammation, aiding tissue repair and regeneration. Inflammation is a multifaceted process that entails activating immune cells and generating a variety of signaling molecules, including cytokines and chemokines [93–95]. However, excessive or prolonged inflammation can lead to tissue damage and scarring and can also interfere with the repair and regeneration of damaged tissue. Engineered biomaterials can be designed to modulate local inflammation in various ways. These biomaterials can be tailored to discharge anti-inflammatory entities like cytokines or growth factors, which can help mitigate inflammation. The pore size of such materials can significantly influence the degree of immune cell penetration. For instance, the pore size of hydrogels can be manipulated through various strategies, such as tweaking the crosslinking density or employing templating techniques [96]. Pore size can affect multiple properties of hydrogels, including their mechanical strength, permeability and ability to support cell adhesion and proliferation [97]. When it comes to the fact that the smaller pore sizes can create physical barriers that limit the movement of cells, as well as reduce the availability of nutrients and oxygen that cells need to survive and increase [98].

**Figure 3.**
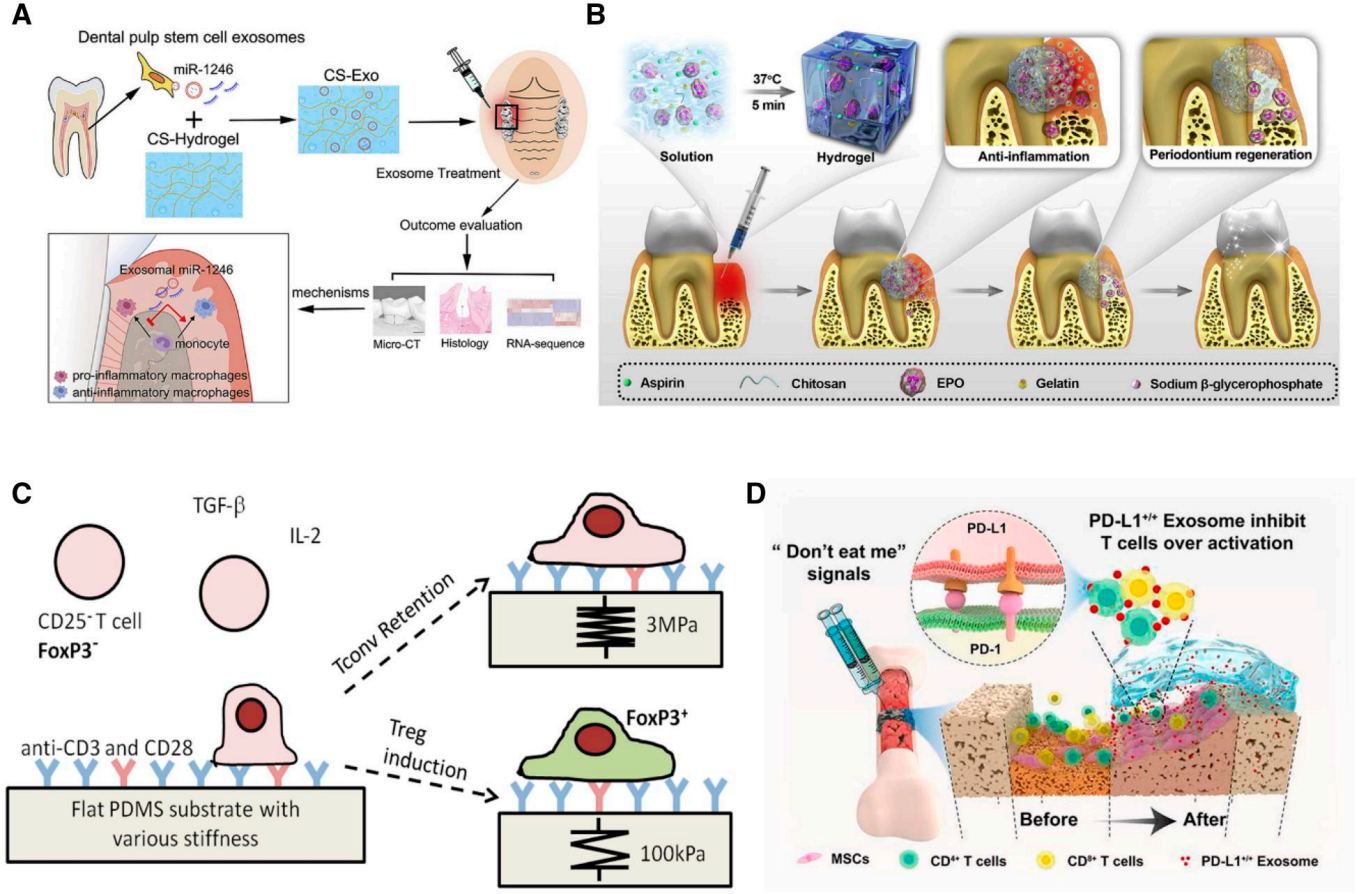
Host response to the transplantation of biomaterials. (**A**) The design of chitosan hydrogel loaded with exosomes secreted by DSCs. Reproduced with permission from Ref. [100], Copyright 2020, Elsevier. (**B**) Schematic illustration of the injectable and thermosensitive hydrogel. Reproduced with permission from Ref. [101], Copyright 2019, Elsevier. (**C**) The design of PDMS substrates with appropriate stiffness for forming treg cells. Reproduced with permission from Ref. [102], Copyright 2018, Wiley. (**D**) The design of PD-L1 conjugated hydrogel for the suppression of T cell activation. Reproduced with permission from Ref. [103], Copyright 2022, Elsevier.

On the other hand, larger pore sizes in hydrogels can promote immune cell infiltration. The larger pore sizes allow for increased diffusion of molecules and nutrients and create more cell space. Furthermore, larger pore sizes could potentially encourage the development of blood vessels, which would enhance the supply of oxygen and nutrients to the cells [99]. Lin *et al.* designed a chitosan hydrogel encapsulating exosomes from DSCs to treat periodontitis by immunoregulating macrophage polarization [100]. The formulated hydrogel can suppress the pro-inflammatory polarization (M1) of macrophages while fostering the pro-healing polarization (M2) of macrophages (Figure 3A). Hydrogels can additionally serve as drug reservoirs to attract DSCs from host tissue and guide their development for successful tissue regeneration. Yang *et al.* engineered an injectable and thermosensitive hydrogel to foster periodontal regeneration [101]. The reported hydrogel was loaded with aspirin and erythropoietin, which can prevent bone resorption and reduce local inflammation (Figure 3B). They can suppress the activation and proliferation of immune cells, reducing inflammation. Lee *et al.* reported the design of cell niches with appropriate stiffness to maximize the production of Treg cells from naïve T cells [102]. The designed PDMS substrates with a stiffness of 100 kPa can effectively induce the formation of Treg cells (Figure 3C). Liu *et al.* [103] developed a hydrogel conjugated with PD-L1 to suppress local inflammation and promote fracture healing. The obtained hydrogel can effectively suppress the activation of T cells only in the local transplantation sites without any systematic immunoinhibitory side effects (Figure 3D).

### Effects of biomaterial degradation on therapeutic outcomes

Biomaterial degradation refers to the process by which biomaterials break down or are broken down by the body over time [104–106]. The degradation of biomaterials can affect therapeutic outcomes in tissue engineering, as the rate and extent of degradation can influence the behavior of cells and the overall effectiveness of the therapy [107]. The degradation rate of biomaterials must be carefully balanced, as both too fast and too slow degradation can compromise therapeutic outcomes [108, 109]. Among all biomaterials used for the delivery and therapy of DSCs in tissue engineering and regenerative medicine, polylactic acid (PLA) is a biodegradable polymer widely used as a scaffold material in tissue engineering [110, 111]. If PLA degrades too slowly, it may hinder tissue regeneration by occupying space that new cells need to infiltrate and grow.

Conversely, if it degrades too quickly, the scaffold may lose its mechanical strength before the new tissue fully matures, potentially leading to collapse or insufficient support. Moreover, collagen is a naturally occurring protein with excellent biocompatibility and biodegradability [112–114]. Its degradation is mainly controlled by enzymatic processes, such as those mediated by matrix metalloproteinases [115–117]. If the collagen degrades too slowly, fibrous tissue may develop around the implant, causing inflammation and impeding the proper functioning of the engineered tissue. If it degrades too quickly, the cells may not have enough time to migrate, proliferate and differentiate, resulting in poor tissue integration.

### Clearance of biomaterials *in vivo*

The clearance of biomaterials *in vivo* is a crucial aspect of their biocompatibility and safety, as it determines how the body processes and eliminates these materials after they have fulfilled their therapeutic purpose [118, 119]. Various factors can influence the clearance of biomaterials, including their chemical composition, physical structure, surface properties and degradation behavior [120]. Biomaterials can be designed to be biodegradable, meaning the body can break them down into smaller molecules that can be safely metabolized or excreted [107, 121]. The rate and extent of biomaterial clearance *in vivo* can vary because of its chemical composition, physical structure and surface properties. They can be classified into three major types in DSCs-mediated tissue engineering and regenerative medicine: nanoparticles, microparticles and hydrogels. The clearance of nanoparticles is strongly influenced by their size, shape, surface charge and surface modifications. Nanoparticles featuring hydrophilic coatings, like polyethylene glycol, are less prone to swift elimination by the reticuloendothelial system and can hence maintain their presence in the bloodstream for extended durations [122–124]. Nanoparticles can be cleared from the body through various routes, including renal excretion (for smaller particles), hepatobiliary clearance (for larger particles), or uptake and degradation by immune cells (especially for particles recognized as foreign by the immune system). Microparticles are more extensive than nanoparticles and can be cleared through biodegradation, phagocytosis by immune cells and excretion via the lymphatic system. For example, microparticles made of poly (lactic-co-glycolic acid) are biodegradable and can be gradually broken down into smaller molecules that can be metabolized or excreted [125–127]. For other polymers, such as PGA (polyglycolide) and PLA (polylactide), the acidic degradation products can cause adverse effects by lowering the pH and inducing an inflammatory response. But most of their products are small molecules that can enter the tricarboxylic-acid cycle or are metabolized by the kidneys. They are excreted as carbon dioxide and water or via urine. Most inorganic particles in devices are elements required for homeostasis and the continuance of physiological processes. Of the following elements, Mg, Zn, Fe, Si, Ge, W and Mo, the most concerning ones are Mg and Si due to their degradation product, hydrogen gas that can damage the surrounding tissues [107, 121–123]. The clearance of hydrogels depends on their degradation behavior, which can be influenced by hydrolytic or enzymatic degradation, swelling and dissolution. For example, hyaluronic acid-based hydrogels can be degraded by hyaluronidases, enzymes naturally present in the body and the resulting degradation products can be safely metabolized or excreted [128–130].

## Conclusion and perspectives

In summary, we comprehensively review DSCs from biomaterial design to host response. Based on their applications, biomaterials can be designed into different types of scaffolds, microspheres and hydrogels. They can be further tailored through engineering methodology to mimic the interactions between DSCs and natural ECM. Engineered biomaterials significantly overcome the limitations of natural biomaterials, thereby improving the outcomes in tissue engineering and regenerative medicine. However, the designed biomaterials must combine physical, biological and mechanical properties while providing the necessary mediators and signaling to appropriate targets to induce tissue regeneration. Another aspect to be focused on is host response after the transplantation of biomaterials. Engineered biomaterials should have good immunoregulatory properties, fast degradation and easy clearance. There is an emerging need to design more and more tools, such as chemistry, materials and biology, to construct better biomaterials to support cell fate determination and tissue regeneration.

Further research is needed to understand biomaterials’ optimal properties and designs to maximize their potential for modulating local inflammation and supporting tissue repair and regeneration before translating *in vitro* and *in vivo* results into clinical trials. Understanding the optimal degradation rate and extent of biomaterials still needs further improvement to maximize their potential for tissue repair and regeneration. Finally, the optimal clearance rate and volume of biomaterials are required to minimize potential adverse effects and maximize their potential for tissue repair and regeneration.
